# Editorial: Use of quantitative systems pharmacology pipelines to bridge *in vitro* and *in vivo* results in drug discovery

**DOI:** 10.3389/fsysb.2023.1291610

**Published:** 2023-09-22

**Authors:** Federico Reali, Attila Csikász-Nagy, Gianluca Selvaggio

**Affiliations:** ^1^ Fondazione the Microsoft Research—University of Trento Centre for Computational and Systems Biology (COSBI), Rovereto, Italy; ^2^ Faculty of Information Technology and Bionics, Pázmány Péter Catholic University, Budapest, Hungary; ^3^ Cytocast Hungary Kft, Budapest, Hungary; ^4^ Pharmetheus AB, Uppsala, Sweden

**Keywords:** quantitative systems pharmacology (QSP), mathematical modelling, model informed drug development, chemical reaction networks (CRNs), artificial intelligence, mechanism of action (MOA), drug discovery

The drug development process is notoriously costly and time-consuming, with a high attrition rate of drug candidates due to unforeseen toxicities or lack of efficacy. Over the past decades, the progressive introduction of quantitative approaches in the modern drug discovery and development process helped mitigate the attrition risks, improving the outcome across different therapeutic areas. However, the design process and consequently the success rate still have room for improvements (Waring et al., 2015; Smietana et al., 2016).

Quantitative systems pharmacology (QSP) has emerged as a promising approach to combine mathematical modeling, experimental data, and biological knowledge to simulate and predict the behavior of drugs within the context of a living organism by integrating systems biology and pharmacometrics approaches (Sorger et al., 2011). QSP can address the challenges faced by pharmaceutical industries from a holistic point of view by incorporating multi-level descriptions of biological phenomena and drug interactions as reviewed by Verma et al. in this Research Topic. The authors present three case studies that illustrate how QSP modeling can help answer specific questions and support decision-making in early drug development. The case studies are an agent-based model of chemotherapy-induced diarrhea and its prediction from organoid experiments; a hybrid model of myocardial infarction and human ventricular progenitor cell therapy; and a parsimonious model of immuno-oncology and the interplay of tumor inhibition, regulatory T cells, and effector T cells. Verma et al. also discuss some of the barriers and facilitators for the successful application and adoption of QSP modeling in the pharmaceutical industry and regulatory agencies.

QSP can bridge the gap between systems biology and pharmacology at different phases of drug discovery as Szalai and Veres and Sommariva et al. showcase in this Research Topic. QSP methods and models can help in identifying new drug targets and determining the mechanism behind them, as well as leveraging these mechanisms to explore the interplay between mutations and drug effects. The review by Szalai and Veres discusses the application of perturbation gene expression profiles in drug discovery. High-throughput gene expression measurements are one of the most frequently used data acquisition methods for such a systems-level analysis of biological phenotypes. However, the correct, mechanistic interpretation of transcriptomic measurements is complicated by the fact that gene expression changes can be both the cause and the consequence of altered phenotype. Perturbation gene expression profiles can help to overcome these problems by directly connecting the causal perturbations to their gene expression consequences.

On the other hand, Sommariva et al. present a mathematical model for studying the effects of mutations and drugs on colorectal cancer (CRC) cells. The authors use chemical reaction networks to describe the signal transduction during the G1-S transition phase in CRC cells. The paper shows how to simulate the effects of loss or gain of function mutations on genes such as KRAS and PTEN, and how to model and optimize the dosage and combination of drugs that target the mitogen-activated protein kinase (MAPK) pathway, such as Dabrafenib and Trametinib. The results are validated using literature data and compared with other approaches, and possible extensions and limitations of the model are discussed.

In recent years, notable advancements have also emerged at the intersection of QSP and machine learning (ML) with the potential to further enhance the predictive power of QSP models or speed up their development. In this Research Topic, Mavroudis et al. use machine learning and mechanistic modeling to predict the plasma exposure of small molecules in early drug discovery. The authors propose a novel framework that combines machine learning (ML) to predict pharmacokinetic (PK) and physicochemical (PC) parameters from molecular structure, and mechanistic models (compartmental-PK and PBPK) to predict plasma exposure using the ML-derived parameters. The authors test their framework on simulated and rat experimental PK data and compare different ML algorithms, molecular representations, and distribution models. Mavroudis et al.’s framework can achieve adequate exposure predictions for most scenarios and can increase the efficiency and accuracy of PK model selection. They also highlight the limitations and challenges of using ML-driven parameters and *in vitro* clearance in PBPK modeling, and how ML approaches can be followed by more conventional pharmacometrics approaches for model refinement.

The articles in this Research Topic explore how QSP methodologies are assisting drug design and development by overcoming obstacles and driving innovation within the field. As highlighted by Verma et al., QSP modeling can be a tool for enhancing drug discovery and development by providing mechanistic insights, hypothesis testing, and outcome prediction. Szalai and Veres discusses the application of large-scale perturbation gene expression profile datasets in the drug discovery process, covering mechanisms of action identification, drug repurposing, pathway activity analysis, and quantitative modeling Sommariva et al. demonstrate how mathematical models can be used as a tool for the *in silico* evaluation of different targeted therapies for colorectal cancer. Mavroudis et al. show how ML frameworks can enable early PK prediction in drug discovery and help prioritize compounds for further evaluation.

Through QSP, researchers can explore various scenarios, optimize dosing regimens, and assess the potential efficacy and safety of drugs before entering clinical phase studies. QSP can help in foreseeing possible shortcomings and drawbacks of compound candidates and offering a quantitative framework to predict how drugs interact within complex biological systems. With the exponential increase in biological data and the constant improvements of modeling methods and algorithms, we are getting closer to reaching a stage when QSP will serve as an essential piece in any drug discovery pipeline. Pharma companies have already started to partner with startups and consultants offering such services, but in our view, soon it will be a general trend to have a dedicated QSP division in most pharma industries.
